# Mobile Application for Ulcer Detection

**DOI:** 10.2174/1874120701812010016

**Published:** 2018-06-29

**Authors:** Luay Fraiwan, Jolu Ninan, Mohanad Al-Khodari

**Affiliations:** 1 Electrical and Computer Engineering Department, Abu Dhabi University, Abu Dhabi, 59911, United Arab Emirates; 2 Biomedical Engineering Department, Jordan University of Science and Technology, Irbid, 22110, Jordan

**Keywords:** Diabetic Foot Ulcer (DFU), Infrared Thermal (IRT) Camera, Red Green Blue (RGB), Mean Temperature Difference (MTD), Thermal Imaging System, Ischemia

## Abstract

**Purpose:**

The number of patients who are suffering from diabetes nowadays is increasing significantly. In some countries, the percentage of population who suffer from this disease can reach up to 20%. Diabetic patients have to deal with their medical conditions and any further complications that this disease may cause. One of the most common conditions is the Diabetic Foot Ulcer (DFU). The early detection of these ulcers can help and may save the life of diabetic patients.

**Methods:**

This work proposes a mobile application for the detection of possible ulcers using a smart phone along with a mobile thermal camera (FLIR ONE). The proposed system captures thermal images of the feet from the thermal camera. The app that identifies ulcers was built using Android studio. The images were acquired to the Samsung S6 smart phone using the FLIR ONE SDK. Image processing techniques were deployed based on Open CV Library. The procedure of detecting possible ulcers was implemented based on analyzing the thermal distribution on the two feet. The developed application compares the difference between the temperature distribution on the two feet and checks if there is a Mean Temperature Difference (MTD) greater than 2.2^o^C (the value which indicates a possible ulcer development).

**Results:**

The system was tested under simulated conditions by heating different locations of the subjects’ feet to different temperature ranges; one image with temperature less than 2.2^o^C and another three images with temperature greater than 2.2^o^C. The system has successfully identified possible ulcer regions along with an image showing the location of the possible ulcers.

**Conclusions:**

This work is a very first step in developing a complete mobile thermal imaging system that can be validated clinically in the future.

## 1 INTRODUCTION

According to the World Health Organization (WHO), diabetes affected 422 million adults worldwide as reported in 2014, and this number is expected to increase [1]. DFU is a symptom of Type 2 diabetes at its advanced stages [2]. Normally around 15% to 25% of diabetic patients suffer from foot complications at a later stage of the disease [3]. These complications are normally attributed to the peripheral ischemia, foot ulceration, and infection [4, 5]. Foot ulcers are mainly caused by peripheral neuropathy, which in turn affects the ability of the patient to sense and feel his foot. As a result, any injury in the foot of the diabetic patient can go unnoticed [6]. Some of the signs of foot ulcers include abundant callus formation, fissures, blisters, and elevated temperature of the ulcer’s region. Normally, these symptoms are analyzed and checked by physicians to make the diagnosis [7]. Most cases of DFU’s are too late to recover the condition, and patients lose their foot or a large portion of it. Therefore, it is crucial to look for early prevention of DFU using early detection techniques to help save the lives of diabetic patients [8]. DFU can be avoided or delayed if adequately treated at an early stage. Currently, the assessment of such foot complications is done frequently by clinicians through analyzing blood circulations, plantar foot pressure, and foot neuropathy [9, 10]. Moreover, specialist clinicians usually assess lower extremity vascular status using Doppler ultrasound. This allows the possibility of getting accurate analysis regarding the current situation of foot ulcers and its risks [11]. However, patients are forced to go for frequent visits to doctors for diabetic foot assessment, which is considered intrusive and costly. In addition, self-assessment is considered difficult because it depends on the knowledge of patients with this disease, and on the usage of medical equipment. The treatments for such complications are commonly associated with therapeutic footwear, foot education, and normal foot care [2]. For example, a modified walking apparatus is used to provide consistent pressure relief at the diabetic patient’s foot. Thus, the prevention of more developed stages of current foot complications situation can be maintained and even healed [12].

Several studies indicate that temperature change is a clear precursor to DFU [3, 21, 22]. However, the temperature differences for DFU regions are small and hard to monitor and determine using analog means. The process of determining ulcerous regions in a diabetic foot with the help of an infrared thermometry has gained ground and has piqued the interest of several researchers [13, 14].

Early detection techniques work best when the diabetic patient applies these techniques immediately after experiencing discomfort/pain is his/her foot. DFU causes extreme pain and discomfort while walking. The patient will have a time frame of one to two weeks before the DFU turns into a full-blown health issue [2, 8]. Thermal infrared thermometry can be recorded using cameras that can capture mid wave or long wave infrared light. The latter is cheaper and can cover wider ranges of temperature. The mid wave infrared thermal cameras are more accurate and therefore better used for medical purposes [15]. However, for home use, the long wave infrared thermal camera can be considered perfect. An example of the long wave infrared thermal camera is the FLIR ONE [16]. Studies have been conducted on preventive techniques for DFU but without using an android compatible FLIR ONE device. Those studies comprise of long, and non-user friendly methods of detection. Color images were used to determine the presence of ulcers and its healing process, but the ulcers would already have to be at their advanced stages [17]. Temperature, humidity, and pressure were measured, by another study, across the soles of the foot by carefully placing the sensors using a smart shoe to determine foot inflammation, which is an indicator for DFU [18]. The sensors send data to the mobile application *via* Bluetooth and a server helps to process the data. A study proposed the combination of digital photography with infrared thermography to acquire the color image and infrared thermometer [7]. FLIR SC305 handheld device was used in some studies to study the early detection of DFU; comprising of a huge apparatus that is not portable, and the images had to be exported to a computer for analysis, run on a server based system from which the results are obtained - making it a long and tedious process [3, 19]. Another study discussed various techniques of DFU prevention using infrared thermal camera [20]. Robust acquisition protocol for early preventive measures for DFU detection has also been proposed as a viable solution [13]. Studies verify that a temperature difference of 2.2 degree Celsius or 4 degree Fahrenheit is a clear indication for the presence of DFU in diabetic patients [3, 21, 22]. Another feasibility study conducted by Fraiwan *et al.* [26] for a Matlab mobile detection system utilizes a Matlab based approach to the early detection of DFU problem. The difference between the current study and this study is the methodology and the outcome of both studies. The current study aimed at implementing a standalone mobile application for the detection of diabetic foot ulcers under simulated patient conditions. It was completely implemented using Andriod Studio, open CV image processing Library, and FLIR ONE SDK. The application was a standalone system

Today, technologies exist that can help determine the presence of ulcers in a diabetic foot. Most of these technologies, however, are large, non-portable, expensive, sometimes invasive, and require an expert physician. This work aims at constructing a standalone Java based mobile thermal imaging system that can be used by diabetic patients to identify possible ulcerous regions in their feet or any other location.

## 2 MATERIALS AND METHODS

The proposed system consists of two major parts: the first part is a thermal image acquisition system that is a smart phone along with a mobile thermal camera, while the second part is a standalone mobile application. The complete system implementation comprises three major steps: image acquisition, image processing, and results display (image interpretation). These steps are described in detail in the following sections.

### 2.1 Thermal Image Acquisition System

The thermal image acquisition system consists of two components: Samsung S6 smart phone and FLIR ONE mobile thermal camera. The FLIR ONE camera is made of two sensors, one is a Lepton thermal sensor, and the other is a visible CMOS sensor. The visible image is overlaid on the thermal image to show the edges on the thermal image producing a final MSX blended image by the FLIR ONE IRT (infrared thermography) camera. The image depicts the hot and cold regions of the area that the camera captures [16]. The camera is based on the principle of blackbody radiation where each body emits its own thermal infrared light or thermal energy, which can be seen only when viewed through the thermal infrared region of the light spectrum [23]. Smart applications using FLIR ONE IRT Camera have been proposed for finding temperature readings in electrical based systems. The FLIR ONE IRT camera can measure high temperature values of up to 120 degrees Celsius. The camera can work continuously for up to an hour after which it needs to be recharged. Moreover, it does not drain the phone’s battery while connected to it. FLIR ONE IRT camera provides an excellent base for thermal image acquisition. The FLIR ONE IRT camera (android) is compatible with various android devices. FLIR ONE also provides a smart application that enables the capturing of IRT images, which are usually used for measuring the temperature of electrical devices. However, they can also be used for medical applications with an accuracy of up to 0.1 degree Celsius. The FLIR ONE device requires some time for charging. The device is simple to use based on plug and use ideology [16].

The used camera is based on the blackbody radiation principle, where an object in the infrared region is attributed by the energy or temperature emitted from the body [24]. However, the reading can be affected by light reflected from the object or its surroundings. Hence, it is best to take the images in a controlled environment where the temperature is set to room temperature and the light is set to a very dim environment. A cold background behind and underneath the object is mandatory. The background may be a concrete wall or a cold towel, and the object can be placed on a surface where the temperatures from the object and from the surface have minimal heat transference. If the image is to be captured by a single user, a non-reflective material such as velvet can be placed around the leg as it can act as a cold surface (background). This simple DIY (Do It Yourself) can be done at home. The distance between the object and the camera must be at least 12 to 16 cm. In addition, there should be no hot objects in the background such as an electrical switch, current carrying wire, hot food, computer, etc. Acquiring a clean image is of utmost importance.

The ulcer was simulated by heating a small metallic object (coin) and pressing it against the foot for heat transference. The heat transference was carefully monitored. The temperature difference on the foot was carefully monitored to have a region of 2.2 degree Celsius temperature difference. The thermal image is captured when the desired temperature difference is achieved.

The smartphone standalone application requires loading the thermal image from the phone’s gallery. The FLIR ONE app provides thermal readings with an accuracy around 0.1 degree Celsius.

### 2.2 Image Processing

A standalone smartphone system with image processing capabilities is made possible with OpenCV and FLIR ONE SDK. OpenCV is a computer vision library used for simple computer vision and image processing techniques on various platforms [25]. It is recommended to use an older version for better support and documentation. OpenCV module was imported and linked to the Android application. The Integrated Development Environment (IDE) used for Android programming was Android Studio. Although there were many other well-known IDEs, Android Studio was selected, as it is Java based and easy to use.

Fig. (**1**) illustrates the complete image acquisition and image processing steps in detail. The acquired thermal image consists of a normal color image as well as the radiometric data embedded within it. There are two sides of the obtained thermal image: the blended MSX image, which is an RGBA image, and the Thermal Radiometric Kelvin, which provides the required thermal radiometric data. The radiometric data is extracted from the thermal image. FLIR provides this support for developers who use FLIR ONE products. FLIR ONE SDK was used to access the radiometric data embedded within the thermal image. FLIR ONE SDK features were invoked to access the radiometric data. The path of the acquired image in the gallery was passed to the SDK and the radiometric data were obtained. The radiometric data is in the format known as Little Endian. Hence, a one-dimensional (1D) data was acquired for the thermal image. The 1D data was converted to 2D data to perform computer vision and image processing techniques to acquire accurate results. The number of values or pixels held by the radiometric data is 320 (Rows) times 240 (Columns), which is 76,800 data values. However, the data was scaled to get a matrix with values ranging from 0 to 255. These values for the radiometric data were obtained in Kelvin times one hundred. A single channel grayscale image can be constructed and shown when displayed. To understand the displayed image, the data in the grayscale image ranges in a color scale of 0-255. The zero value represents the lowest temperature from the actual radiometric data, and the 255 value represents the highest temperature. Moreover, the scaled values were used to extract portions of the image following an image segmentation procedure. Applying a binary threshold to the grayscale helps in segmenting the feet of the patient from any background structures. This is based on the idea of temperature variation between the feet segments (Hot) and the background (Cold). A threshold of 149 was applied on the grayscale image to extract the feet segments. The binary image was then eroded to remove any noise (wrongly classified background segments) that might be present. The segments were then dilated to recover the foot area that might have been lost because of the eroding process. Now feet segments were considered as the background, whereas the suspected hottest region was the foreground. The hottest region was extracted using another binary threshold of 244 for the grayscale. Although the hottest region has been identified, verification must be applied to decide whether this was an ulcerous region or not. The decision was taken based on the temperature variation between the suspected hottest region and the normal feet region. The threshold values of 149 and 244 were selected after meticulously studying various scenarios involved in early detection of DFU such as shape, size, and region of the suspected ulcerous region of the foot by using the process as described in the previous subsection. The threshold values depict the suspected region with highest variation in temperature as compared to the rest of the foot. In addition, the normal feet region was analyzed without the suspected ulcer region. The mean temperature of both regions was then calculated and prepared for further analysis. For this, the radiometric data that was previously saved was used, as the mean temperature observed was only for the pixels that depicted the required region. The difference between both extracted segments provided the needed outcomes to decide whether the case was ulcerous, which was an increase of more than 2.2 ^o^C, or normal feet temperature variations.

Usually to determine whether the foot is ulcerous, the mean temperature of the feet segment, which is represented without the hottest region, is subtracted from the mean temperature of the hottest region. The Mean Temperature Difference (MTD) is then calculated. If the MTD is 2.2 ^o^C less, the foot is normal, and no ulcer is detected. However, if the MTD is above 2.2 ^o^C, then an ulcer is present. Moreover, false positives and false negatives results are obtained if the feet region and the hottest region are below 25 ^o^C, or if the feet region and the hottest region are above 50 ^o^C.

This represents the worst-case scenario with a maximum temperature that the human foot can have. If either of these conditions is triggered, then no ulcer is detected. This is under the assumption that the background is completely cold. On the other hand, the BGR image was extracted from the original thermal image. Upon reading the image, OpenCV was set to output the image in the BG R order representing the Blue, Green, and Red channels. Colors were used to signify the ulcerous region, if present. For example, blue represented the foot devoid of ulcer and red represented the ulcerous region. The background of the thermal image was removed from the BGR image to obtain only the feet segment. This was done by first converting the BGR to grayscale image. The acquired image is shown in Fig. (**5**). The stand-alone smartphone application automatically applies the developed algorithms and threads on the radiometric data to represent it as a 2D grayscale image. The image shown for the user is a color image of a range from 0 to 255, which represents the variations of the temperature on the acquired image between the background and the feet (foreground). Next, a threshold is applied to the greyscale image to filter out the low values and set them to zero. This works in the same way as a high pass filter. The segmentation procedure is shown in Fig. (**6**), where two thresholds were used to extract the feet from the background and to obtain the suspected hot region from the plantar feet. The application provides the results to the user as images including the hot, hottest, and the hot minus hottest segments. Moreover, within the application, mean temperature values for both feet, including the hot minus hottest region and the suspected hottest region segments, are represented to the user. Moreover, this helps in removing the background and acquiring a binary image, which represents only the feet segment. The result combines a BGR image of the foot without the background with the ulcer region that has been highlighted in red. The red indicates the critical region of the foot. If ulcer is not present, the result will be the BGR foot without the background and without the highlighted red region.

The application was implemented using two threads: one main thread that provides the user interface as shown in Fig. (**2**), and a background thread that includes the main functionality of the system. This latter thread is illustrated in Fig. (**3**). The background thread includes the image processing part as listed in Fig. (**4**). The background thread protects the main thread from being clogged up with processing techniques, which helps in providing a smooth flowing user experience.

## 3 RESULTS

The result shown to the user is the overall view of his feet with the help of a BGR image, as shown in Fig. (**7**). The blue color on the screen represents the cold segments on the image, and the red region represents the hot segment, which indicates the occurrence of DFU. The Mean Temperature Difference (MTD) is provided to the user, which is the difference between the suspected hottest region and the feet region. If the MTD is more than 2.2 ^o^C, the application highlights the BGR image with red color on the suspected ulcerous region.

Four test images were used to test the algorithm developed using OpenCV software. The first image is for a non-diabetic feet patient with no abnormalities. The other three images are with simulated ulcer on different regions. The four test images are shown in Figs. (**8**, **9**, **10**, and **11**) where the mean temperature values for the feet segments (hot minus hottest) are 33.2 ^o^C, 36.9 ^o^C, 35.5 ^o^C, and 35.3 ^o^C, respectively. The suspected hottest region mean temperature values are 35.0 ^o^C, 39.4 ^o^C, 38.0 ^o^C, and 37.7 ^o^C, respectively. If an ulcer occurs, the application automatically highlights the region with a red color over the BGR blue feet segment image.

The MTD for the first test image shown in Fig. (**8**) is 1.8 ^o^C, while the MTD values for the rest of images containing a simulated ulcer region are 2.5 ^o^C, 2.5 ^o^C, and 2.4 ^o^C, respectively. Table **1** depicts the observed values for the four test images.

## 4 DISCUSSION

The proposed mobile thermal imaging system represents a new promising approach to detect possible ulcers in the diabetic patients’ feet, which can facilitate for them how to deal with such a medical case. The new mobile thermal camera FLIR ONE was used for this purpose along with an application that was developed to identify possible ulcers. The mobile application, in its first form, was tested successfully under simulated conditions, which included heating 4 different areas in the feet to check the ability of the proposed application to identify certain elevated temperature regions. One image was tested for a temperature difference less than 2.2 degrees Celcius and the other three images were tested with temperature increased slightly greater than 2.2 degrees Celcius. The system has successfully identified the hottest region and if it represents a possible ulcer or not. It is worth to keep in mind that this application is not a diagnosis tool; instead, it is just an indicator for DFU’s. The patient must immediately consult his/her physician for a complete checkup. Early prevention techniques can help save the lives of diabetic patients and ease their pain by preventing the disease before the need for a cure.

The application was made as a standalone system that requires computation time to perform image processing within the smartphone’s processor. The smartphone processor can handle the required computation with a computation time of 5 to 6 seconds. The performance period is acceptable in this stage considering that complete privacy of the user is achieved. However, this delay must be reduced to improve performance.

Security features can be added at any point. Since the application runs entirely on the android device, security can be enabled by encrypting the phone. This makes sure that the device is secure. There is no loss of data since network access is not required. Hence, there is no sharing of data, thereby preserving complete user privacy.

## CONCLUSION AND FUTURE WORK

In conclusion, a smartphone application for ulcer detection has been implemented with the use of FLIR ONE IRT camera. Its compact, portable, and lightweight nature gives it an added advantage for home applications to help determine DFU at its early stages. The smartphone application using thermal techniques can be applied to several real-life problems. Moreover, with the introduction of built-in thermal camera in smartphones such as CAT S60, this eliminates the need for a separate IRT device.

## Figures and Tables

**Fig. (1):**
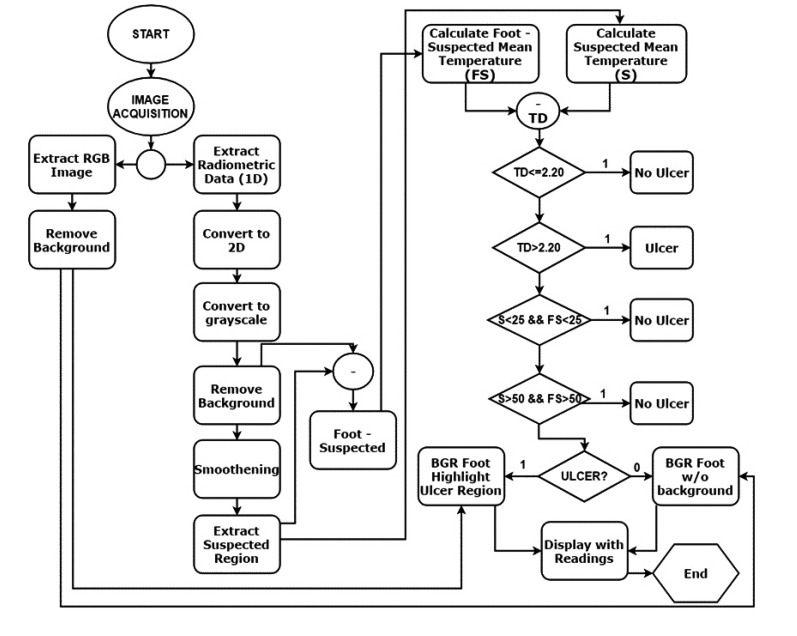
The overall system.

**Fig. (2):**
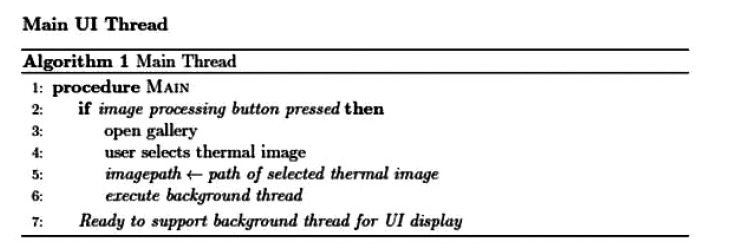
Main UI.

**Fig. (3):**
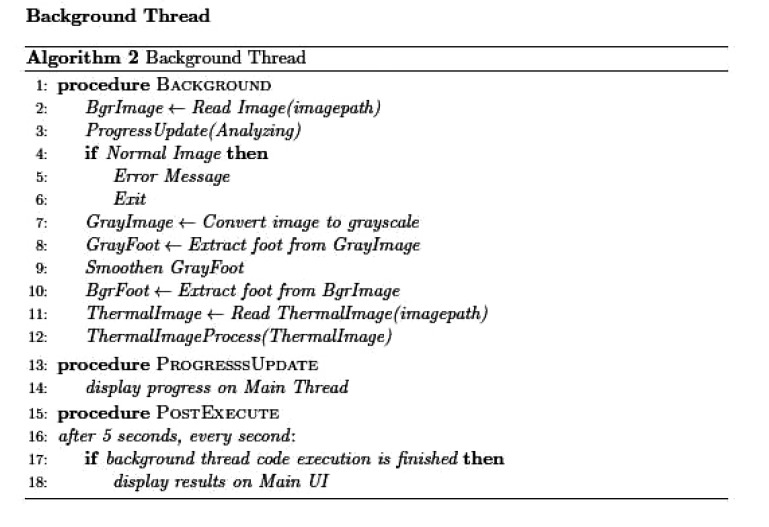
Background thread.

**Fig. (4):**
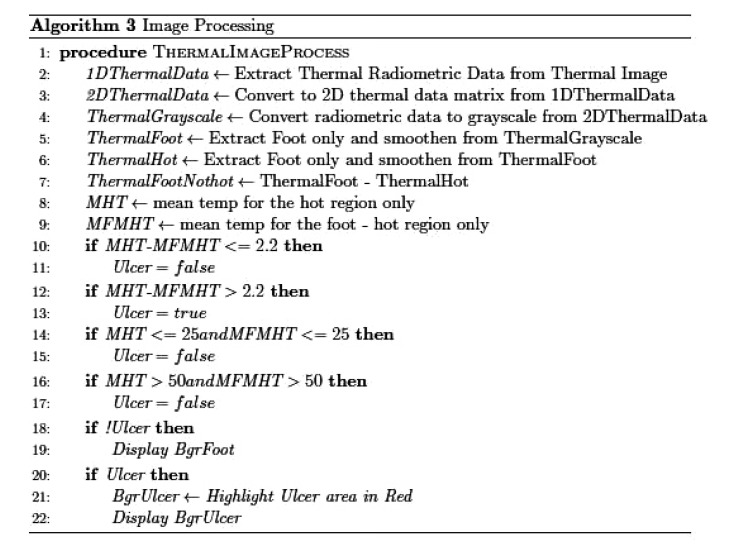
Image processing.

**Fig. (5):**
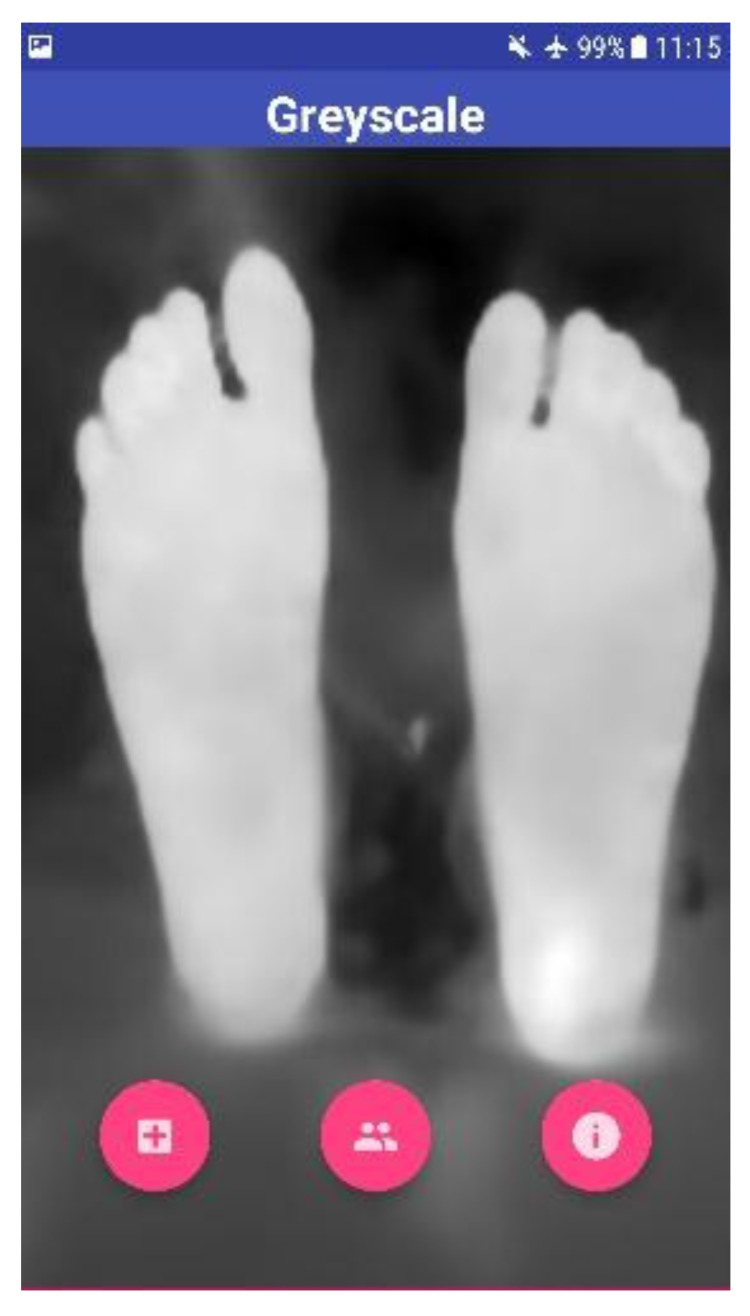
Grayscale Image.

**Fig. (6):**
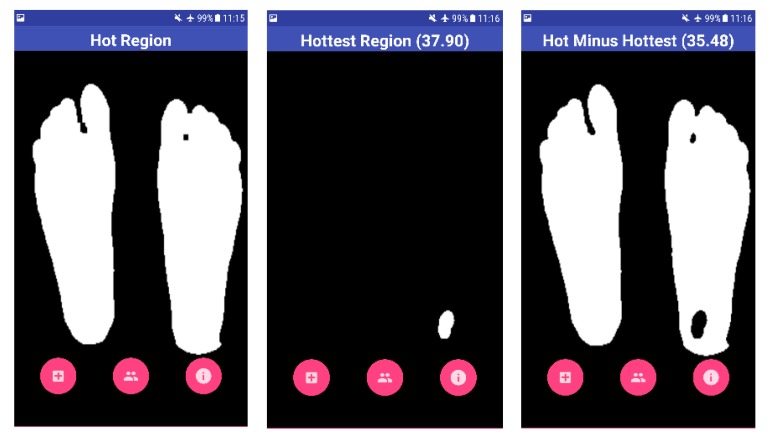
Feet (Left), Hottest Region (Center), Foot - Hottest Region (Right).

**Fig. (7):**
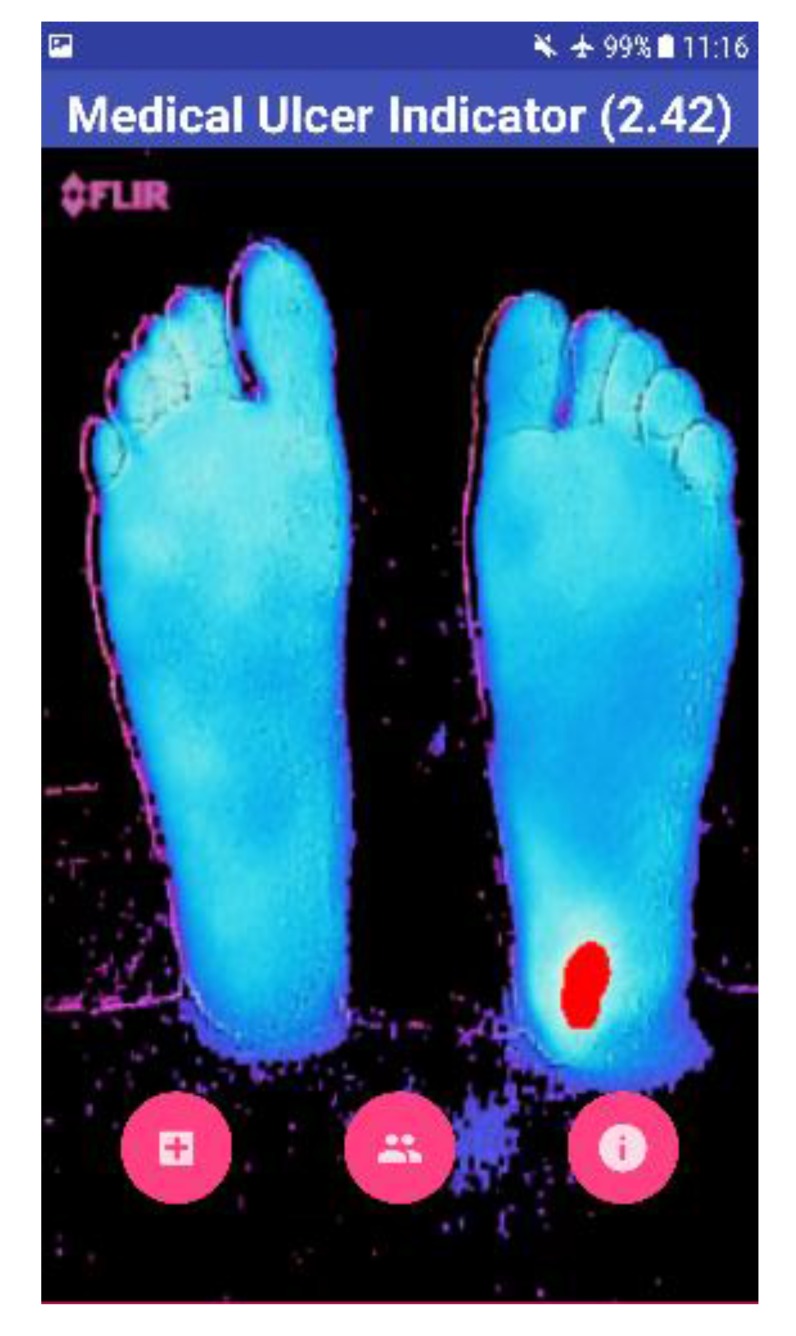
Result (Ulcer Detected).

**Fig. (8):**
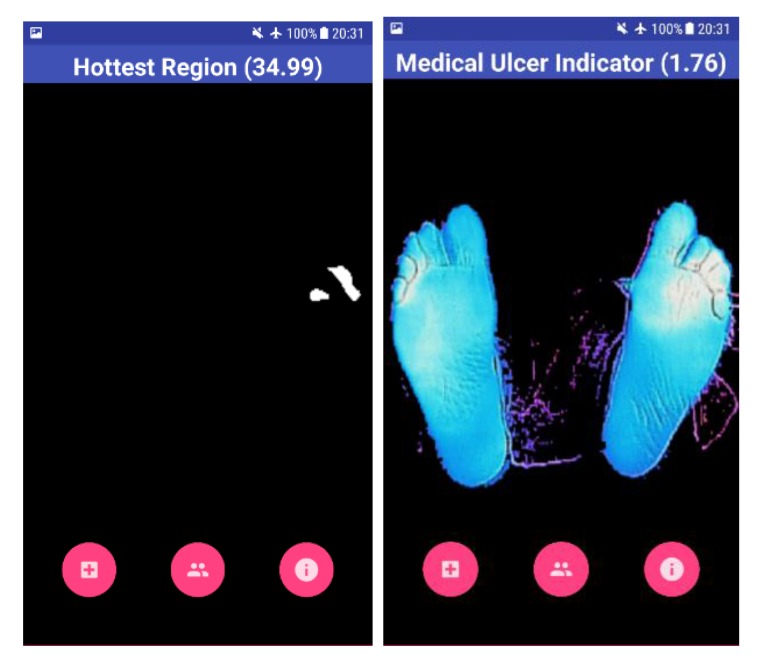
Test Image 1.

**Fig. (9):**
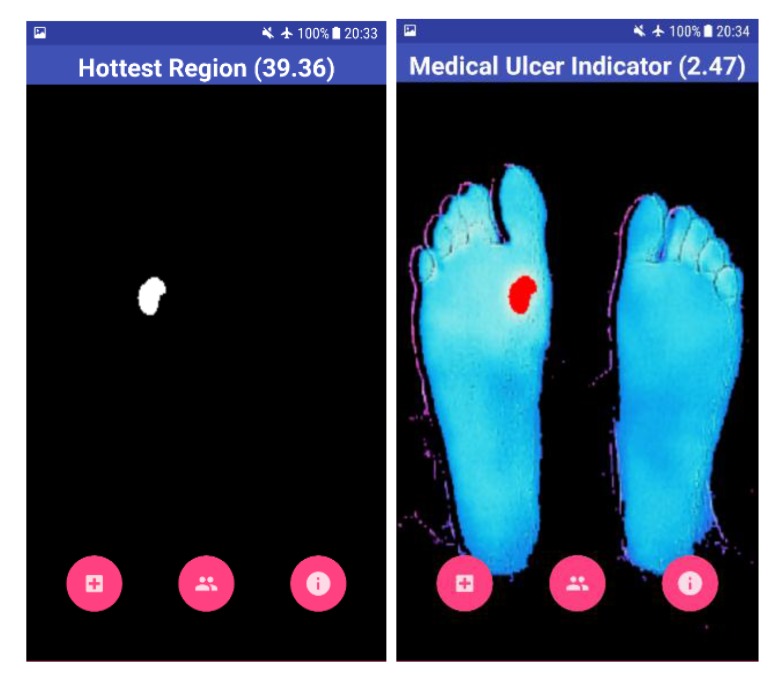
Test Image 2.

**Fig. (10):**
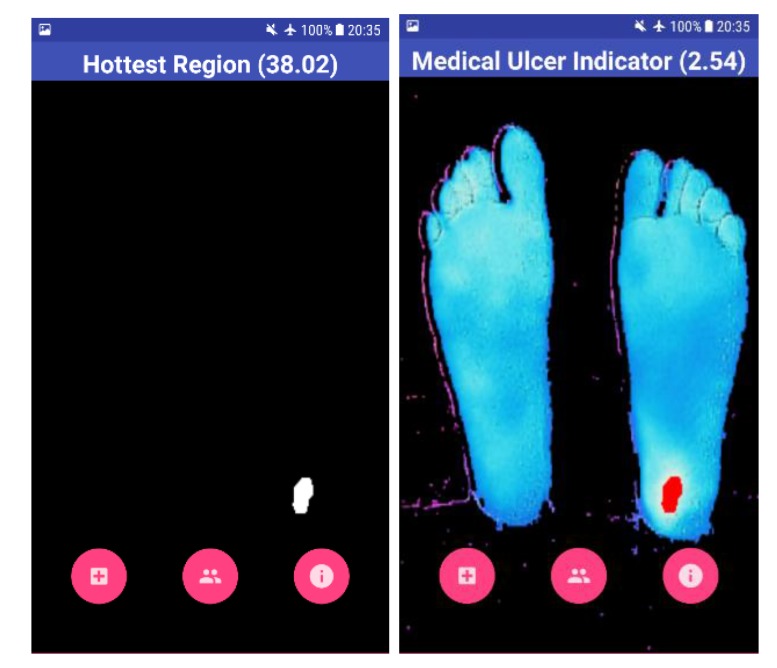
Test Image 3.

**Fig. (11):**
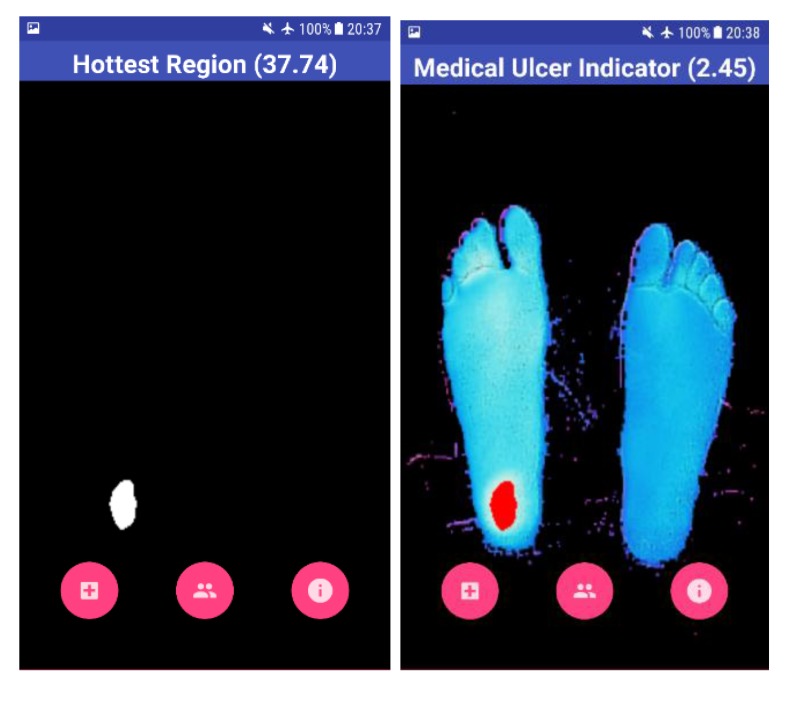
Test Image 4.

**Table 1:** Temperature values measured from suspected regions tested on 4 different images.

	Hot minus Hottest Mean Temperature	Suspected Hottest Region Mean Temperature	Mean Temperature Difference
Test Image 1	33.2 ^o^C	35.0 ^o^C	1.8 ^o^C
Test Image 2	36.9 ^o^C	39.4 ^o^C	2.5 ^o^C
Test Image 3	35.5 ^o^C	38.0 ^o^C	2.5 ^o^C
Test Image 4	35.3 ^o^C	37.7 ^o^C	2.4 ^o^C
